# The outcomes and prognostic factors of the patients with unplanned intensive care unit readmissions: Erratum

**DOI:** 10.1097/MD.0000000000011673

**Published:** 2018-07-20

In the article, “The outcomes and prognostic factors of the patients with unplanned intensive care unit readmissions”,^[1]^ which appeared in Volume 97, Issue 26 of *Medicine*, the author affiliations should appear as:

^a^Departments of Orthopedics and Trauma, Chi Mei Medical Center, Tainan, Taiwan

^b^Department of Respiratory Therapy, Chi Mei Medical Center, Liouying, Tainan, Taiwan

^c^Department of Intensive Care Medicine, Chi Mei Medical Center, Liouying, Tainan, Taiwan
